# Gallium-Indium-Tin Eutectic as a Self-Healing Room-Temperature Liquid Metal Anode for High-Capacity Lithium-Ion Batteries

**DOI:** 10.3390/ma15010168

**Published:** 2021-12-27

**Authors:** Weldejewergis Gebrewahid Kidanu, Jaehyun Hur, Il Tae Kim

**Affiliations:** 1 Department of Chemical and Biological Engineering, Gachon University, Seongnam-si 13120, Gyeonggi-do, Korea; weldatmail@gmail.com; 2 Department of Chemical Engineering, Ethiopian Institute of Technology-Mekelle (EIT-M), Mekelle University, Mekelle 231, Tigray, Ethiopia

**Keywords:** room-temperature liquid metals, liquid metal nanoparticles, self-healing, gallium-indium-tin eutectic, lithium-ion battery

## Abstract

Owing to their intrinsic properties, such as deformability, high electrical conductivity, and superior electrochemical performance, room-temperature liquid metals and liquid metal alloys have attracted the attention of researchers for a wide variety of applications, including portable and large-scale energy storage applications. In this study, novel gallium-indium-tin eutectic (EGaInSn) room-temperature liquid metal nanoparticles synthesized using a facile and scalable probe-ultrasonication method were used as anode material in lithium-ion batteries. The morphology, geometry, and self-healing properties of the synthesized room-temperature liquid metal nanoparticles were characterized using scanning electron microscopy (SEM) and transmission electron microscopy (TEM) with energy-dispersive X-ray spectroscopy (SEM/EDS and TEM/EDS). The synthesized room-temperature liquid metal nanoparticles delivered a specific capacity of 474 mAh g^–1^ and retained 77% of the stable reversible capacity after 500 galvanostatic charge-discharge cycles at a constant current density of 0.1 A g^–1^. The high theoretical specific capacity, combined with its self-healing and fluidic features, make EGaInSn room-temperature liquid metal nanoparticles a potential anode material for large-scale energy storage applications.

## 1. Introduction

Finite deposits of fossil fuels and problems arising from their excessive use are driving researchers to develop devices for sustainable energy storage. Graphite, the conventional anode in commercial lithium-ion batteries (LIBs), has a low capacity (~372 mAh g^–1^) and a low discharge potential, which make it unable to fulfill increasing energy and power density demands [1,2,3,4,5]. Therefore, low-cost, high-power density, high energy density, reasonable coulombic efficiency, and long cycle-span batteries are urgently needed [5,6]. Metal nanoparticles have attracted the attention of researchers for a wide variety of applications, including plasmonics, catalysis, and sensors [7,8,9]. Nanostructures can enhance the capacity and rate capability of battery materials by shortening the ion diffusion paths [10]. Owing to their intrinsic properties, such as deformability, high electrical conductivity, and superior electrochemical performance, room-temperature liquid metals (RTLMs) and liquid metal alloys have attracted attention for use in portable and large-scale energy storage applications [11,12]. RTLMs offer unique metallic and fluidic properties; specifically, when deformed, they remain soft and highly conductive [13,14]. Compared to conventional LIBs, which contain a considerable amount of inert materials, RTLMs are believed to deliver a higher specific capacity because they intrinsically possess a higher amount of electroactive materials [12]. Hence, RTLM rechargeable batteries have become straightforward alternative energy storage devices. Gallium (Ga), a post-transition metal with a melting point of 29.8 °C, is one of the most interesting elements in the periodic table [15]. Ga has a water-like bulk viscosity of 1.99 mPa·s (vs. 1 mPa·s for water) [1,15]. Yamaguchi et al. reported the reversible size control of gallium liquid metal nanoparticles (LMNPs) through ultrasonication [7]. Liquid metal alloys have intrinsic self-healing properties that stem from their liquidity and surface tension. Recently, Wu et al. reported an “ultra-high-long cycle life” RTLM alloy of Ga and Sn (Ga:Sn = 88:12 wt%) as a high-capacity anode material for LIBs [16]. The theoretical specific capacities of the Ga and Sn anodes are ~769 mAh g^–1^ and ~993 mAh g^–1^, respectively. As the capacity of Sn is higher than that of Ga, alloying Ga with a small fraction of Sn could, theoretically, enhance the net capacity. The specific capacity of Sn is also close to that of In (1012 mAh g^–1^). Hence, alloying Sn, Ga, and In in a eutectic proportion could produce a RTLM alloy with better electrochemical properties. Moreover, the electronic conductivity of Sn (7.17 × 10^6^ S m^−1^) is larger than that of In (1.3 × 10^6^ S·m^–1^) and Ga (7.1 × 10^6^ S·m^–1^). Hence, the addition of Sn to the GaIn liquid metal is expected to improve the electrical performance of the RTLM GaInSn eutectic (EGaInSn). In addition, the electronegativity of Sn (1.96) is close to that of Ga (1.81) and In (1.78). Moreover, alloying Ga with In and Sn in appropriate amounts lowers the melting point. For instance, mixing Ga, In, and Sn in a eutectic ratio of 62:22:16 wt% results in a RTLM alloy with a melting temperature of ~15 °C. This low-temperature liquid alloy is capable of self-healing at room temperature [16]. Therefore, advantages and synergistic effects on the overall electrochemical performance in LIBs are expected by incorporating Sn into the GaIn eutectic. The EGaInSn bulk liquid metals were also converted into LMNPs. The EGaInSn LMNP electrodes can benefit from nanostructuring, which enhances the electrochemical performance of electrode materials by shortening the metal ion and electron diffusion paths. Furthermore, the high active surface area in nanoparticles enables faster electrochemical kinetics [10,17,18]. In contrast, Sn (15.34 Euro/kg) is less expensive than Li (88.76 Euro/kg), Ga (213 Euro/kg), and In (262 Euro/kg); Sn is ~6, ~14, and ~17 times less costly than Li, Ga, and In, respectively [12]. Ga and Ga-based liquid metal alloys are also less toxic and have negligible vapor pressure at room temperature [14,18]. As a result, EGaInSn has been considered as a potential substitute for toxic and volatile mercury in liquid metal thermostats [19]. To the best of our knowledge, ternary EGaInSn LMNPs have never been used as electrode materials for LIBs. Hence, in this study, the electrochemical performance of EGaInSn LMNPs was investigated as a potential self-healing anode material for LIBs.

## 2. Materials and Methods

### 2.1. Chemicals and Materials

Gallium-indium-tin (62 wt% Ga, 22 wt% In, and 16 wt% Sn) RTLM eutectic (99.99% metal basis) and Li metal foil (99.9% metal basis, 0.75 mm thick) were purchased from Alfa Aesar (Ward Hill, MA, USA). Sodium dodecyl sulfate (SDS) was purchased from Sigma-Aldrich.

### 2.2. EGaInSn LMNP Synthesis

The EGaInSn LMNPs were synthesized using a simple and facile probe ultrasonication method. Specifically, 50 mg of EGaInSn was dropped into 1 mL of *N*-methyl-2-pyrrolidone (NMP) solvent and then 1 mg of SDS surfactant was added, before ultrasonication with a Sonics VCX-750 Vibra-Cell Ultrasonic Liquid Processor for 2 h. Each pulse was 40 s long, followed by 55 s off. The power, frequency, and amplitude of the ultrasonication processor were maintained at 750 W, 20 kHz, and 20%, respectively. To prepare the slurry, 40 mg of super P carbon black (CB) and 10 mg of polyvinyl difluoride (PVDF) binder (200 mg in 5% PVDF in NMP) were added to the LMNP suspension in a mass ratio of 5:4:1, and 2 mL of NMP was added to maintain a uniform slurry during mixing. The mixture was homogenized using a vortex mixer. Next, 40 µL of the slurry was drop-casted onto a Cu foil and dried at 45 °C under vacuum, calendered, weighed, and calculated. The active material weight was 0.5–1.0 mg.

### 2.3. Material Characterization

The structure of the EGaInSn LMNP electrodes was characterized using high-resolution X-ray diffraction (SmartLab, Rigaku, Japan), and the electrode materials were hermetically sealed with Kapton-type polyimide foil. The morphology, geometry, and chemical composition of the synthesized materials were characterized using scanning electron microscopy (SEM, Hitachi S-4700), transmission electron microscopy (TEM, JEOL 2100), and scanning transmission electron microscopy (STEM) techniques in combination with energy-dispersive X-ray spectroscopy (SEM/EDS and TEM/EDS). SEM was used to investigate the self-healing nature of the LMNP electrodes.

### 2.4. Electrochemical Performance of the LMNPs

CR2032-type coin cells were assembled in an argon-gas-filled glovebox. The galvanostatic charge–discharge cycles and rate performance tests of the EGaInSn LMNP electrodes were tested using an automatic battery cycling system (WonATech, WBCS3000, Seoul, South Korea) in constant current mode, and data were acquired using an IV MAN photovoltaic analyzer. The electrochemical performance was tested using both glass fiber (GF) prefilters (Merck Millipore, Ireland) and polyethylene (PE) separators using 120 µL of electrolyte (1 M LiFP_6_ in diethylene carbonate/ethylene carbonate, 1:1, *v/v*). Cyclic voltammetry (CV) and electrochemical impedance spectroscopy (EIS) tests were performed on a ZIVE MP1 workstation (WonATech, Seoul, South Korea). The CV measurements were conducted at a scan rate of 0.05 mV s^–1^ in a potential window of 0.01–1.5 V vs. Li/Li^+^, and the EIS spectra of the electrodes were obtained before and after cycling in the frequency range of 100 kHz to 100 mHz, with a perturbation potential of 5 mV.

## 3. Results and Discussion

### 3.1. Synthesis and Characterization of as-Prepared EGaInSn LMNPs

**Figure 1:**
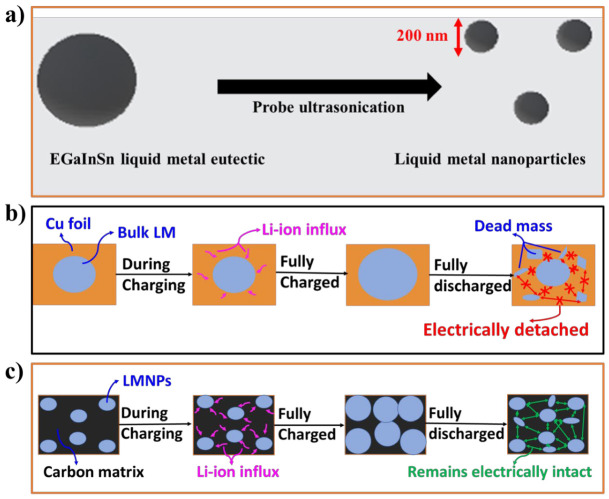
Schematic illustrations of (**a**) the synthesis process of EGaInSn LMNPs by probe sonication, (**b**) the delamination of typical electrodes made up of bulk liquid metal alloys, and (**c**) the self-healing property of LMNP electrodes.

Gallium has a melting temperature of 29.76 °C. Alloying gallium with indium and tin in a weight ratio of 62:22:16 produces a eutectic alloy with a melting point of 10.7 °C. The melting point can be varied by changing the composition (Table S1). For example, the weight ratio 68:22:10 for Ga, In, and Sn, respectively, slightly increases the melting point to 11 °C [14]. Regardless, small variations in the weight ratio and melting point of EGaInSn do not appreciably change its chemical and physical properties [14,20]. Reducing the size of the bulk liquid metal into LMNPs reduces the diffusion distance within each particle [21] and improves the capacity and rate performance of the material [10]. Ultrasonication is one of the simplest and most scalable methods for producing LMNPs from bulk liquid metals [7]. Herein, the EGaInSn bulk liquid metal (Figure S1) was converted into LMNPs using probe ultrasonication (Figure 1a). Controlling the shape, size, and structure of LMNPs is essential, as the electrochemical properties and structural features are strongly related [7,22]. Hence, an SDS surfactant was used to prevent the aggregation and coalescence of the LMNPs [7,23,24,25].

Once the LMNPs were homogenized with CB and a PVDF binder, the slurry was drop-casted onto a Cu foil and dried at 45 °C. The formation of EGaInSn LMNPs by probe ultrasonication is schematically illustrated in Figure 1a. The schematic illustration in Figure 1b describes the structural delamination (the partial fragmentation of the electrode material) due to the huge volume expansion or detachment of the active materials from the current collector [26,27]) of bulk EGaInSn RTLM during repeated cycling. The dead mass in the bulk liquid metal electrode remains electrically disconnected. This leads to rapid capacity fading and battery failure. However, using LMNPs instead of their bulk form prevents such electrode disintegration through the self-healing ability of the LMNPs, as schematically illustrated in Figure 1c. Electrodes with EGaInSn LMNPs remain electronically connected, which makes them prospective electrode materials for LIBs with high capacity, stability, and long cycle life. The green arrows in the schematic in Figure 1c illustrate the electrical connections due to the presence of a conductive carbon matrix, whereas the absence of this matrix is indicated by the red arrows in Figure 1b in the bulk liquid metal electrodes.

**Figure 2:**
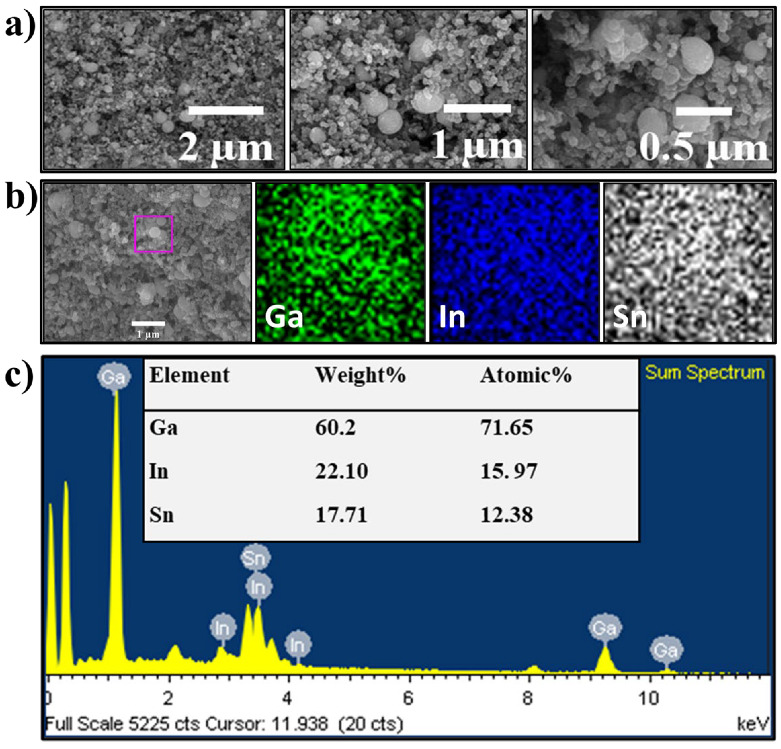
(**a**) SEM images, (**b**) SEM/EDS mapping, and (**c**) SEM/EDS elemental analysis of the as-prepared EGaInSn LMNP electrode. SEM/EDS mapping and elemental analysis were focused on the pink box in (**b**).

**Figure 3:**
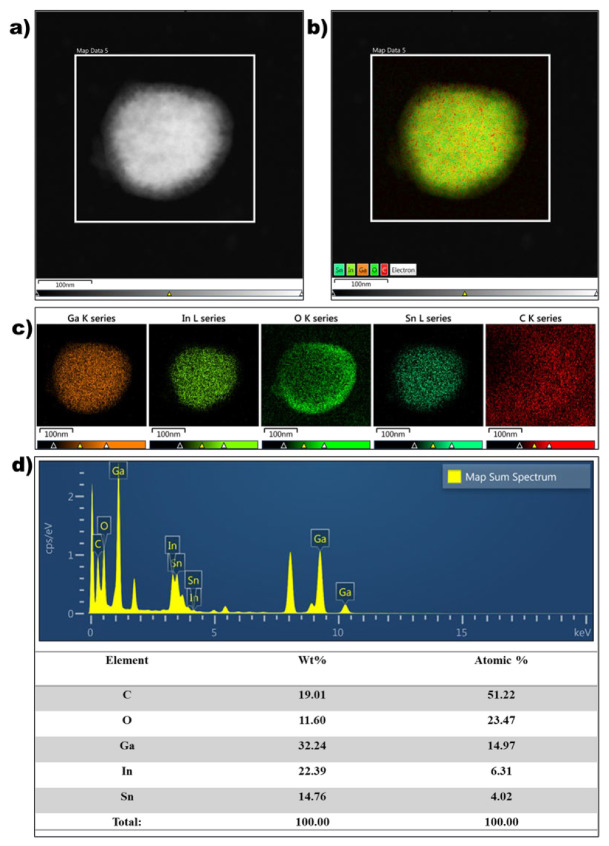
(**a**) TEM electron image, (**b**) STEM image, (**c**) TEM/EDS mapping, and (**d**) TEM/EDS spectra and atomic composition of a single EGaInSn LMNP.

The morphology and microstructure of the EGaInSn LMNPs were studied using SEM and TEM. SEM images of the as-prepared LMNP electrodes showed that the size of the individual LMNPs ranged from 200 to 250 nm (Figure 2a). SEM/EDS images of a single EGaInSn LMNP displayed a uniform elemental distribution of Ga, In, and Sn (Figure 2b). The elemental distribution on a larger scale was also uniform (Figure S2). A weight ratio of 60.2% Ga, 22.1% In, and 17.71% Sn was determined from the SEM/EDS measurements (Figure 2c). Further investigation using TEM revealed that the average size of the individual EGaInSn LMNPs was ~200 nm (Figure 3a and Figure S3), which was consistent with the SEM results. The SEM and TEM images reveal that the EGaInSn LMNPs have a spherical shape, indicating that the alloying process is isotropic (i.e., the growth of the EGaInSn LMNPs is equal in all directions), which results in a uniform geometry and predictable material properties. STEM images and the corresponding EDS mapping of the as-prepared EGaInSn LMNPs (Figure 3b,c) further confirmed the uniform atomic distributions of Ga, In, Sn, O, and C. Notably, the TEM/EDS signal for oxygen was stronger than the other elements on the surface. Considering the fact that Ga and Ga-based alloys instantly form a thin oxide layer when exposed to air [7,23,28], a strong O signal is expected, as the EGaInSn LMNPs were synthesized in air. The elemental compositions from the TEM/EDS analysis of the as-prepared EGaInSn LMNPs also showed consistent atomic and weight compositions (Figure 3d).

### 3.2. Electrochemical Performance of EGaInSn LMNPs and Ex-Situ Characterization

**Figure 4:**
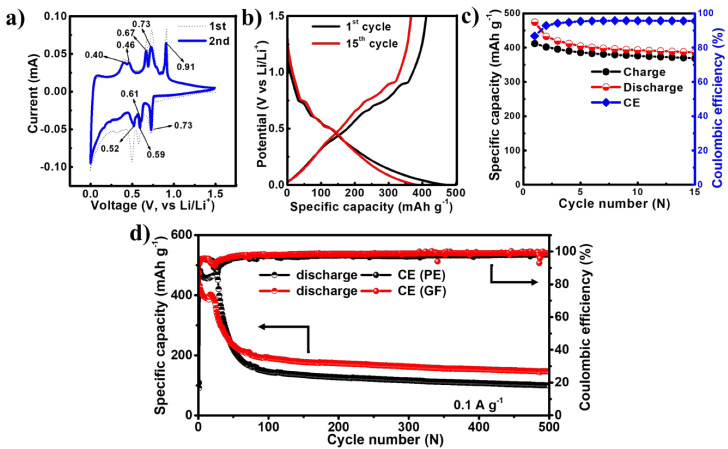
Electrochemical performance of EGaInSn LMNPs with a GF micro separator and PVDF binder: (**a**) CV, (**b**) typical potential profiles, (**c**) galvanostatic charge/discharge profiles at the initial stages measured with a GF separator right after the CV measurements, and (**d**) long term cycling performance of EGaInSn LMNP electrodes at 0.1 A g^–1^ with GF and PE separators.

The electrochemical performance of the EGaInSn LMNP electrodes was tested between 0.01 and 1.5 V (vs. Li/Li^+^) in a CR2032-type coin cell using Li foil as a counter/reference electrode. To study the reversibility and electrochemical properties of the EGaInSn LMNPs, CV measurements were conducted using linear potential scanning at 0.05 mV s^–1^. The first two CV profiles are shown in Figure 4a. The CV profiles showed reversible anodic peaks at 0.4, 0.46, 0.67, 0.73, and 0.91 V, whereas the corresponding reduction peaks appeared at 0.73, 0.61, 0.59, and 0.52 V (vs. Li/Li^+^), respectively. The typical potential profiles of the EGaInSn LMNPs measured immediately after the CV measurements are shown in Figure 4b. The plateaus in the potential profiles appeared where the current peaks in the CV profiles appeared, indicating that the electrochemical reactions were consistent and were due to the formation of different stable phases of the material. The initial constant-current galvanostatic charge-discharge profiles of the cell after CV measurements are presented in Figure 4c. Furthermore, the long-term cycling performance of the cells was investigated. As shown in Figure 4d, two cells were assembled using GF and PE separators. Both cells demonstrated similar electrochemical behaviors. The initial reversible capacity and coulombic efficiency of the cell with the GF separator were 474 mAh g^–1^ and 86%, respectively, and were 481 mAh g^–1^ and 80% for the cell with the PE separator. The initial specific capacities of the cells with GF (474 mAh g^–1^) and PE (481 mAh g^–1^) separators at 0.1 A g^–1^ were 55% and 56% of the calculated theoretical capacity (857.82 mAh g^–1^, see Supplementary Material for the calculations), respectively.

The long-term cycling performance measurements revealed that the cells took ~100 cycles to stabilize, which was also the case in other liquid metal-based alloy batteries such as gallium-indium eutectic [29,30]. After 100 stabilization cycles, the cells operated for more than 500 cycles, retaining 77% and 69% of the stable capacity after 500 cycles for the cells with GF and PE separators, respectively, as summarized in Table S2. The coulombic efficiencies of the cells after 500 cycles were 98% and 99% for the cell with the PE and GF separators, respectively. The CV, potential profiles, and initial cycling behaviors of the EGaInSn LMNP electrodes with a PE separator are also provided in the Supplementary Materials (Figure S4). The long-term cycling stability of the cells was mainly due to the self-healing nature of the EGaInSn LMNPs. Ga-based self-healing LMNPs have a recoverable morphology, which greatly improves the cycling performance [31].

**Figure 5:**
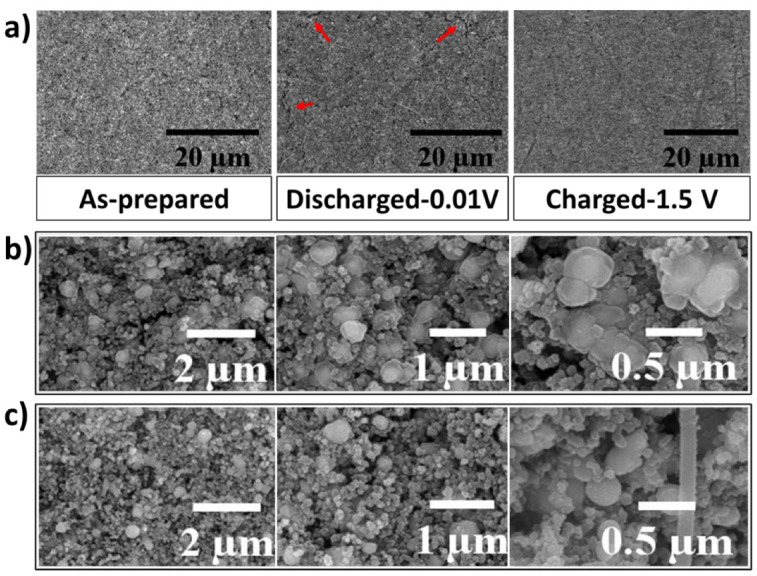
(**a**) Low magnification ex-situ SEM images of as-prepared, discharged (to 0.01 V vs. Li/Li^+^), and charged (to 1.5 V vs. Li/Li^+^) EGaInSn LMNP electrodes, (**b**) high magnification ex-situ SEM images of fully discharged, and (**c**) fully charged electrodes. The charging and discharging of the electrodes were conducted at a current density of 0.1 A g^–1^.

To further study the self-healing behavior of the LMNPs, the cycled electrodes were investigated using ex-situ SEM analysis. The morphology and structural evolution of the as-prepared, fully charged, and fully discharged EGaInSn LMNP electrodes were monitored at high and low magnifications. Figure 5a shows low-resolution SEM images of as-prepared, fully lithiated (discharged to 0.01 V vs. Li/Li^+^), and fully extracted (charged to 1.5 V vs. Li/Li^+^) states of the EGaInSn LMNP electrodes. As seen from the highlighted section of the fully discharged electrode in Figure 5a, the cracks disappeared and the surface became strikingly smoother after fully charging, displaying the self-healing property of the LMNPs. Liquid-metal-based electrodes with self-heling properties are resistant to structural cracks [12,32]. To further reveal the self-healing nature of the EGaInSn LMNPs, detailed ex-situ SEM images were obtained. As shown in Figure 5b, the spherical shapes of the EGaInSn LMNPs flattened, when fully lithiated, indicating the insertion of Li ions into the structures of the liquid metal composites; when fully charged again (Figure 5c), they recovered their original spherical shape, which is consistent with the self-healing properties of the material.

**Figure 6:**
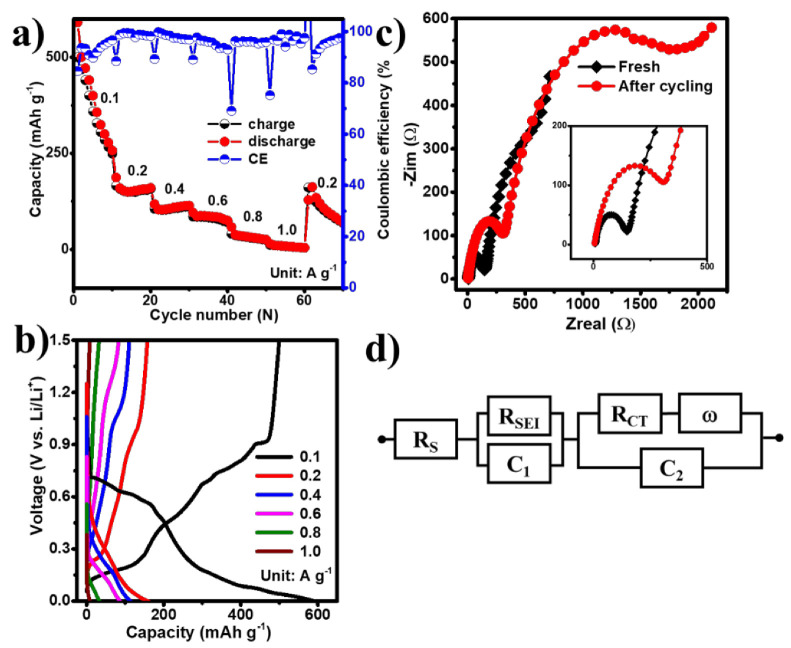
The (**a**) rate performance test, (**b**) typical voltage profiles at different current densities, (**c**) EIS spectra, and (**d**) corresponding equivalent circuit model.

Following the cycling performance tests, the rate capabilities of the EGaInSn LMNPs were studied. The cells delivered reversible discharge capacities of 500, 166, 105, 85, 40, and 12 mAh g^–1^ at current densities of 0.1, 0.2, 0.4, 0.6, 0.8, and 1 A g^–1^, corresponding to coulombic efficiencies of 85%, 88%, 89%, 89%, 96%, and 97%, respectively (Figure 6a,b). In addition, the EIS spectra of the cells were measured before and after cycling. As shown in the inset of Figure 6c, the cycled cell exhibited an increased charge transfer resistance (R_CT_), compared to the fresh cell, which could be attributed to the formation of the solid electrolyte interface (SEI) layer. The electrolyte resistance (Rs), SEI layer resistance (R_SEI_), and R_CT_ values, extracted by fitting the EIS data using the equivalent circuit in Figure 6d, are summarized in Table 1.

**Figure 7:**
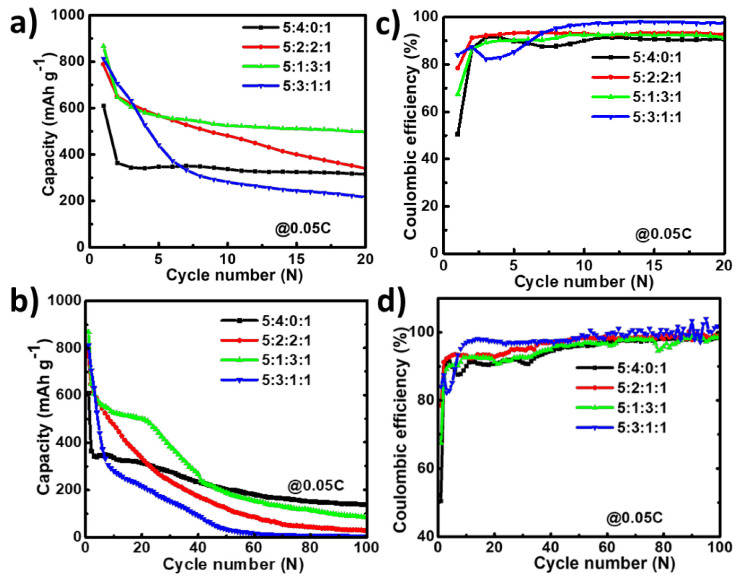
Effect of graphite on cyclic performance and coulombic efficiency of EGaInSn LMNP electrodes in the first 20 cycles (**a**,**b**) and over 100 cycles (**c**,**d**) at a current density of C/20 (0.05 C or 50 mA g^–1^). The cells were operated in a potential window of 0 to 1.5 V vs. Li/Li^+^.

As discussed above, the initial capacity utilization is low compared to the theoretical capacity (less than 60%). Hence, to enhance the capacity, graphite powder was incorporated into the slurry by changing the EGaInSn LMNP:CB:PVDF slurry ratio from 5:4:1 to 5:2:2:1 and 5:3:1:1 (EGaInSn LMNP:CB:Graphite:PVDF), as shown in Figure 7. Graphite has a low density and lubricating effect; hence, it is believed to enhance the electrochemical performance of the EGaInSn LMNPs without significantly affecting the capacity. In agreement with this, the addition of graphite at all proportions (10, 20, and 30 wt%) significantly enhanced the capacity in the early cycles (Figure 7a). However, the cell without graphite was more stable compared to those containing graphite over more cycles, as shown in Figure 7b, which might be due to the exacerbated side reactions as a result of the incorporation of highly conductive graphite at such low current density. The corresponding coulombic efficiencies of the cells are shown in Figure 7c,d. Therefore, the addition of graphite has a great potential to enhance the initial cycling properties. Nevertheless, to achieve long-term cycling stability, further optimization of the electrode fabrication is being conducted and will be reported elsewhere in the near future.

## 4. Conclusions

In this study, room-temperature self-healing EGaInSn LMNPs were synthesized using a simple and scalable probe ultrasonication method. The morphology, geometry, and chemical composition of the synthesized EGaInSn LMNPs were characterized using SEM, SEM/EDS, and TEM measurements. The self-healing ability of the EGaInSn LMNPs was confirmed by ex-situ SEM imaging. The LMNPs were electrochemically tested as anode materials for LIBs with GF and PE separators, and satisfactory results were obtained. Specifically, the EGaInSn LMNPs delivered an initial specific discharge capacity of 474 mAh g^–1^ and a coulombic efficiency of 98.5% at a constant current density of 0.1 A g^–1^. The self-healing anode materials retained 77% of the stable discharge capacity after 500 cycles at 0.1 A g^–1^, and the final coulombic efficiency was 99%. Owing to the fluidic nature at room temperature and the high theoretical capacity of the ternary metal eutectic, further study on EGaInSn LMNPs will lead to high-energy self-healing electrodes for large-scale energy storage LIBs.

## Figures and Tables

**Table 1:** EIS results extracted using the equivalent circuit in Figure 6d.

	R_s_ (Ω)	R_SEI_ (Ω)	R_CT_ (Ω)
Fresh cell	2.20 μ	155.83	132.20
After cycling	22.77	349.94	1106.36

## Data Availability

Supplementary data related to this article can be found at Supplementary Materials.

## Supplementary Materials

The following are available online at https://www.mdpi.com/article/10.3390/ma15010168/s1: Figure S1: photographs of (a) the bulk EGaInSn liquid metal (LM) in NMP, (b) LMNPs after ultrasonication in NMP, and (c) the EGaInSn LMNP slurry on a Cu foil; Figure S2: elemental mapping and SEM/EDS spectra of as-prepared EGaInSn LMNP electrodes; Figure S3: TEM image of as-prepared EGaInSn LMNPs; Figure S4: (a) CV, (b) typical potential profiles, and (c) initial cycling behaviors after CV measurements of the EGaInSn LMNP electrodes using a PE separator; Table S1: Room temperature liquid metals and their melting points; and Table S2: capacity and coulombic efficiency of EGaInSn LMNP electrodes using different separators.
