# Supplementary material for: Gallium-Indium-Tin Eutectic as a Self-Healing Room-Temperature Liquid Metal Anode for High-Capacity Lithium-Ion Batteries
Source: Materials (Basel). 2021 Dec 27;15(1):168. doi: 10.3390/ma15010168 (PMC8746264; doi:10.3390/ma15010168)
Supplement: Supplementary file 1 [file materials-15-00168-s001.zip › materials-1492593-supplementary.pdf]

materials

MDPI

Article

Gallium-Indium-Tin Eutectic as a Self-Healing Room-Temperature Liquid Metal Anode for High-Capacity Lithium-Ion Batteries

Weldejewergis Gebrewahid Kidanu [1,2], Jaehyun Hur [1,*] and Il Tae Kim [1,*]

1 Department of Chemical and Biological Engineering, Gachon University, Seongnam-Si 13120, Gyeonggi-do, Korea; weldatmail@gmail.com
2 Department of Chemical Engineering, Ethiopian Institute of Technology-Mekelle (EIT-M), Mekelle University, Mekelle 231, Tigray, Ethiopia
* Correspondence: jhhur@gachon.ac.kr (J.H.); itkim@gachon.ac.kr (I.T.K.); Tel.: +82-31-750-5593 (J.H.); +82-31-750-8835 (I.T.K.)

Calculating theoretical specific capacities

The theoretical specific capacities of the electrodes were calculated as:

$$C_{sp}^{th} = \frac{nF}{\sum M'} \quad (1)$$

where C_{sp}^{th} is the theoretical specific capacity, n is the number of moles of electrons involved in the reaction, F is the Faraday constant (26.8 Ah g^{-1}), and $\sum M$ is the sum of the molecular weights of the reactants (in grams). The theoretical capacities of pure Ga, In, and Sn were 769, 1012, and 990 mAh g^{-1}, respectively. Hence, the theoretical specific capacity of EGaInSn at a composition of Ga (62%), In (22%), and Sn (16%) was

= 769 × 0.62 + 1012 × 0.22 + 990 × 0.16 mAh g^{-1}

= 857.82 mAh g^{-1}.

Table S1. Room temperature liquid metals and their melting points.

Element/Alloy	wt%	Melting Point (°C)	Reference
**Ga**	100	29.9	
**In**	100	156.6	
**Sn**	100	231.9	[1]
**Eutectic GaSn**	86.5:13.5	20.5	
**Eutectic GaIn**	78.6:21.4	15.3	
**Eutectic GaInSn**	68.5:21.5:10	−19	
**Eutectic GaInSn**	62:22:16	10.7	Used in this study

Table S2. Capacity and coulombic efficiency of EGaInSn LMNP electrodes using different separators.

	Glass Fiber		Polyethylene	
	Cycle number			
	100th	500th	100th	500th
**Capacity (mAh g^{-1})**	192	147	145	100
**Coulombic efficiency (%)**	97.5	99.00	97	98
**Capacity retention (%)**	77		69	

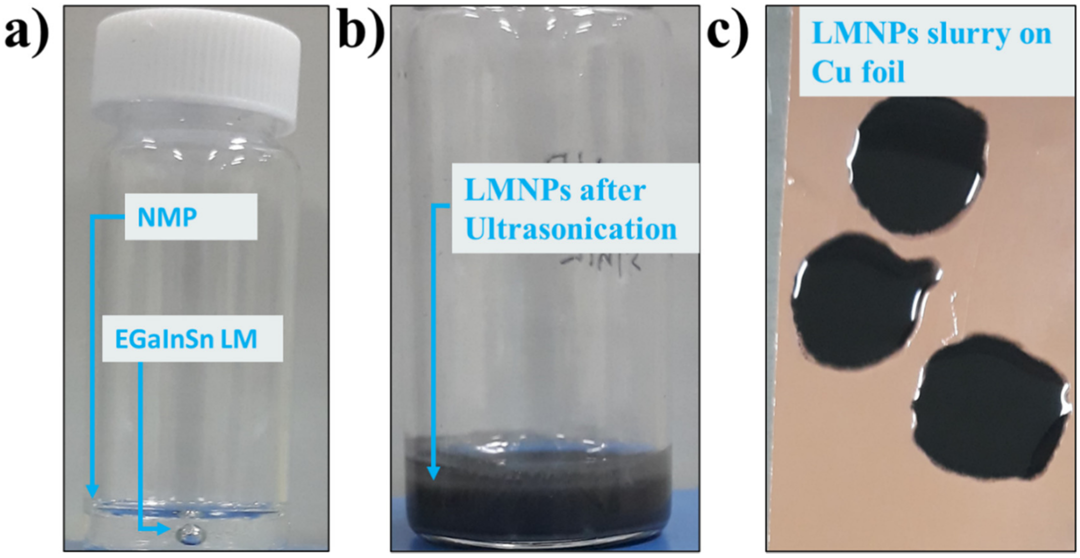

Figure S1. Photographs of (**a**) the bulk EGaInSn liquid metal (LM) in NMP, (**b**) LMNPs after ultrasonication in NMP, and (**c**) the EGaInSn LMNP slurry on a Cu foil.

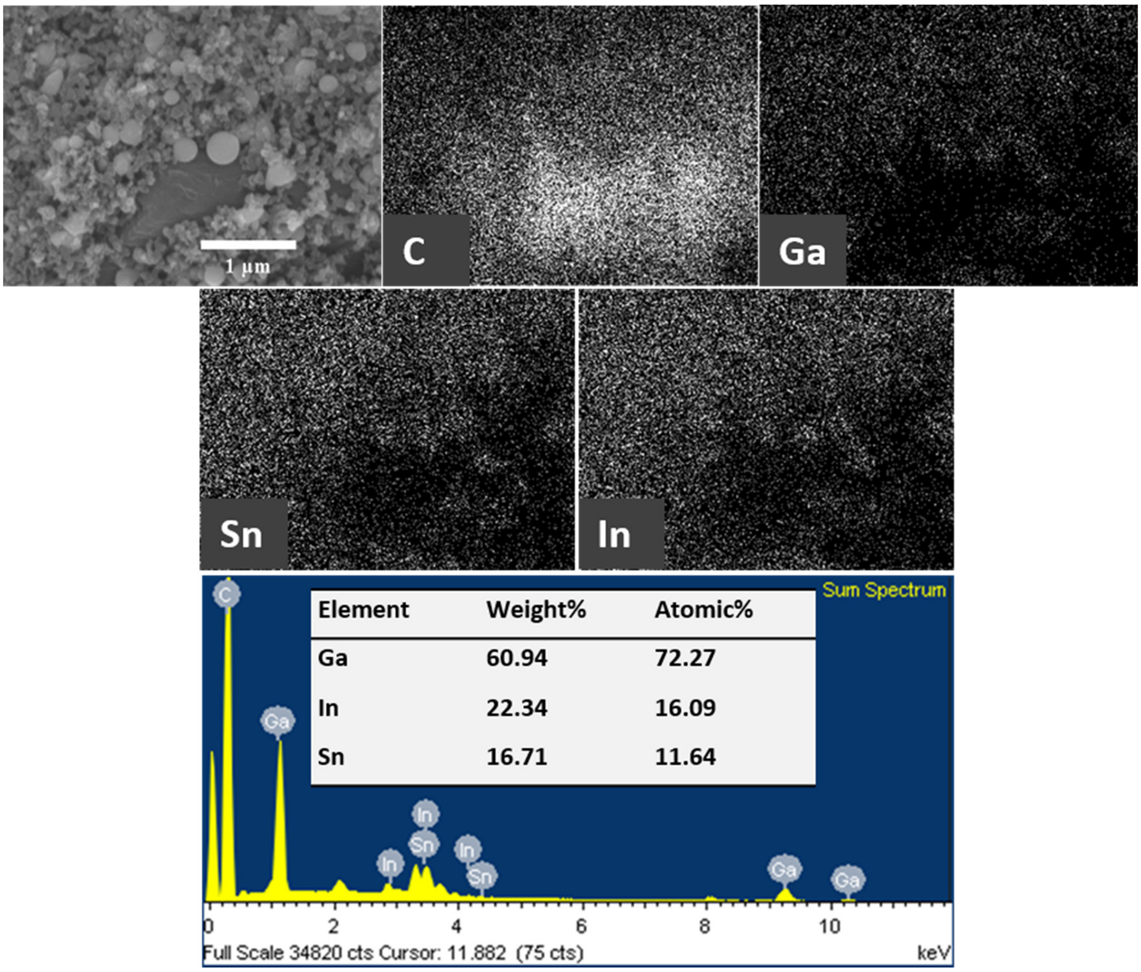

Figure S2. Elemental mapping and SEM/EDS spectra of as-prepared EGaInSn LMNP electrodes.

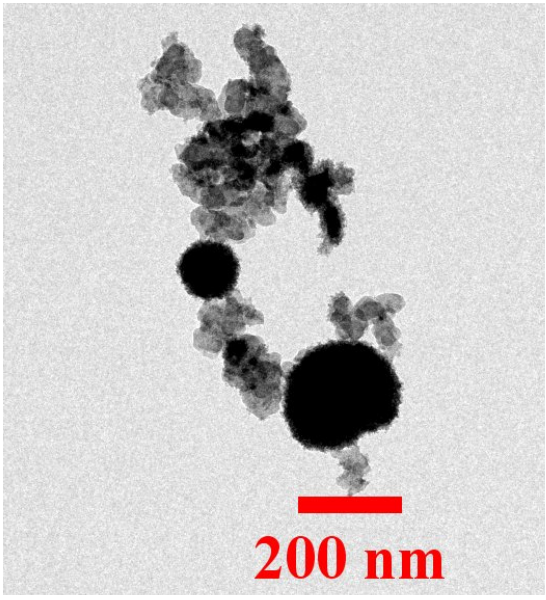

Figure S3. TEM image of as-prepared EGaInSn LMNPs.

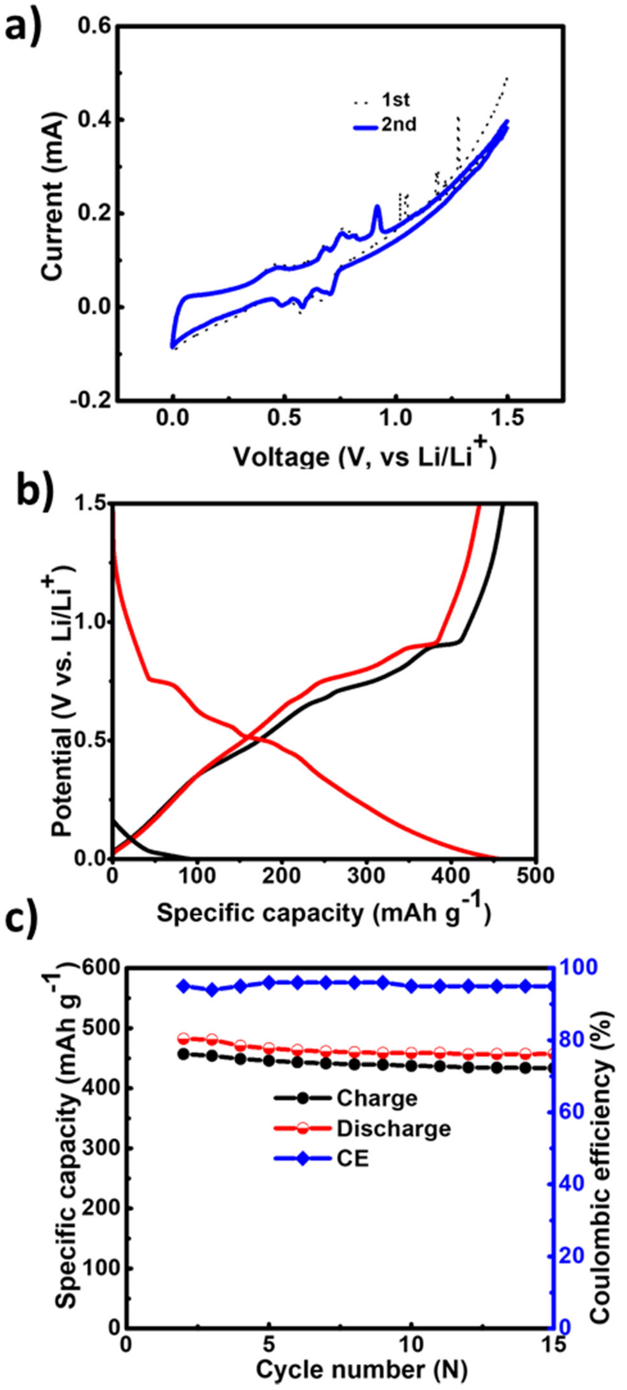

Figure S4. (**a**) CV, (**b**) typical potential profiles, and (**c**) initial cycling behaviors after CV measurements of the EGaInSn LMNP electrodes using a PE separator.

References

[1] M. Song, J. Niu, W. Cui, Q. Bai, Z. Zhang, Self-healing liquid Ga-based anodes with regulated wetting and working temperatures for advanced Mg ion batteries, *J. of Mater. Chem. A*, **2021**, *9*, 17019–17029.
